# *Lactobacillus reuteri* LR1 Improved Expression of Genes of Tight Junction Proteins via the MLCK Pathway in IPEC-1 Cells during Infection with Enterotoxigenic *Escherichia coli* K88

**DOI:** 10.1155/2018/6434910

**Published:** 2018-08-19

**Authors:** Hongbo Yi, Li Wang, Yunxia Xiong, Zhilin Wang, Yueqin Qiu, Xiaolu Wen, Zongyong Jiang, Xuefen Yang, Xianyong Ma

**Affiliations:** State Key Laboratory of Livestock and Poultry Breeding, Ministry of Agriculture Key Laboratory of Animal Nutrition and Feed Science in South China, Guangdong Public Laboratory of Animal Breeding and Nutrition, Guangdong Key Laboratory of Animal Breeding and Nutrition, Institute of Animal Science, Guangdong Academy of Agricultural Sciences, 1 Dafeng 1st Street, Guangzhou 510640, China

## Abstract

Intestinal epithelial barrier damage disrupts immune homeostasis and leads to many intestinal disorders. *Lactobacillus reuteri* strains have probiotic functions in their modulation of the microbiota and immune system in intestines. In this study, the effects of *L. reuteri* LR1, a new strain isolated from the feces of weaning piglets, on intestinal epithelial barrier damage in IPEC-1 cells caused by challenge with enterotoxigenic *Escherichia coli* (ETEC) K88 were examined. It was found that *L. reuteri* LR1, in large part, offset the ETEC K88-induced increase in permeability of IPEC-1 cell monolayers and decreased the adhesion and invasion of the coliform in IPEC-1 cells. In addition, *L. reuteri* LR1 increased transcript abundance and protein contents of tight junction (TJ) proteins zonula occluden-1 (ZO-1) and occludin in ETEC K88-infected IPEC-1 cells, whereas it had no effects on claudin-1 and F-actin expression. Using colloidal gold immunoelectron microscopy, these effects of *L. reuteri* LR1 on ZO-1 and occludin content in IPEC-1 cells were confirmed. By using ML-7, a selective inhibitor of myosin light-chain kinase (MLCK), the beneficial effect of *L. reuteri* LR1 on contents of ZO-1 and occludin was shown to be dependent on the MLCK pathway. In conclusion, *L. reuteri* LR1 had beneficial effects on epithelial barrier function consistent with increasing ZO-1 and occludin expression via a MLCK-dependent manner in IPEC-1 cells during challenge with ETEC K88.

## 1. Introduction

The intestinal epithelial barrier plays an essential role in the host defense against pathogen infection [1]. An impaired epithelial barrier disrupts immune homeostasis and exacerbates inflammation in many diseases, such as postweaning diarrhea stress, enteric pathogen infection, inflammatory bowel disease (IBD), irritable bowel syndrome, obesity, metabolic syndrome, and liver diseases [2–6]. The tight junctions (TJ) between adjacent epithelial cells create a semipermeable barrier that prevents bacteria and other harmful substances from crossing the epithelium [7]. Disruptions of TJ proteins increase the permeability of the epithelial barrier and cause inflammation in the intestine [8], leading to many intestinal diseases. Although antibiotics have been widely used to treat intestinal diseases in past decades, recent studies have demonstrated that antibiotic exposure disrupts both the normal composition of intestinal microbiota and expression of TJ proteins hence damaging intestinal epithelial barrier function [9–11]. All this emphasizes the need to identify safe and effective agents for the treatment of intestinal diseases associated with damage to the epithelial barrier.

*Lactobacillus reuteri* (*L. reuteri*) strains are important members of the commensal microbiota in intestines of humans and other animals and have been demonstrated to regulate the development of the immune system in the gut [12–14]. *L. reuteri* can produce reuterin, which exhibits a broad-spectrum antimicrobial activity against intestinal pathogens [15–17]. In addition, human *L. reuteri* reduces intestinal inflammation by inhibiting the toll-like receptor 4- (TLR4-) nuclear factor *κ*B (NF-κB) signaling pathway [18, 19]. It also improved intestinal epithelial barrier function by promoting cell migration [20]. Previous studies have demonstrated that some probiotics increase the expression of intestinal tight junction proteins through p38/MAPK or myosin light-chain kinase (MLCK) signaling pathways [21]. The latter regulates myosin light-chain (MLC) phosphorylation and then modulates intercellular permeability and apoptosis of intestinal epithelial cells [22, 23]. The strain *L. reuteri* LR1 was isolated from the feces of healthy weaned piglets in our previous study, and its 16S rRNA sequence had been deposited in the GenBank database (accession number KT205306) [24] The *L. reuteri* LR1 showed beneficial effects on intestinal epithelial barrier functions [24]. The effects and underlying mechanisms of *L. reuteri* LR1 on intestinal epithelial barrier function during challenge with enterotoxigenic *Escherichia coli* (ETEC) are, as yet, incomplete.

The objective of this study was to investigate effects and underlying mechanism of *L. reuteri* LR1 on ETEC K88-induced damage of the epithelial barrier function in an *in vitro* model using intestinal porcine epithelial cells.

## 2. Materials and Methods

### 2.1. Bacterial Cultures

*L. reuteri* LR1 was isolated from the feces of a healthy weaned piglet (Duroc × Landrace × Large White), as described [24]. *L. reuteri* LR1 was grown at 37°C for 18 h in MRS broth. ETEC K88 was obtained from the Institute of Veterinary Drug Control of China and grown in lysogeny broth at 37°C for 16 h. Bacterial cells of ETEC K88 and *L. reuteri* LR1 were suspended at the required concentration in Dulbecco's Modified Eagle's Medium (DMEM, Invitrogen, Carlsbad, CA).

### 2.2. Cell Culture

Intestinal porcine epithelial cells (IPEC-1) were a gift from Dr. Guoyao Wu (Texas A&M University). The cells were cultured in DMEM supplemented with 10% inactivated fetal bovine serum (Gibco) and antibiotics (100 U/ml penicillin and 100 *μ*g/ml streptomycin sulfate) at 37°C under 5% CO_2_ in a humidified incubator.

IPEC-1 cells were cultured in Transwell dishes (Corning Life Sciences, Corning, NY) for 21 d as before [24]. Monolayers of IPEC-1 cells were incubated for 6 h in DMEM without serum or antibiotics, in the presence of ETEC K88 (1 × 10^7^ CFU), *L. reuteri* LR1 (1 × 10^8^ CFU), or both, in the upper chamber. Permeability of the IPEC-1 cell monolayers was measured with FITC-dextran (4400 Da, Sigma-Aldrich, St Louis, MO) [24]. IPEC-1 cells were collected for enumeration of ETEC K88, real-time PCR (qPCR), and Western blotting analysis. Six wells per treatment were used, and the results were representative of 3 independent experiments.

### 2.3. Treatment with Inhibitor ML-7

IPEC-1 cells were seeded in 6-well plates (5 × 10^5^ cells per well) and cultured for 24 h. The cells were pretreated with 50 *μ*M ML-7 (a selective inhibitor of MLCK) or vehicle (0.1% DMSO) for 6 h and then treated with *L. reuteri* LR1 (1 × 10^8^ CFU for 6 h) before exposure to ETEC K88 (1 × 10^7^ CFU for 1 h), then IPEC-1 cells were collected for Western blotting analysis. Six wells per treatment were used.

### 2.4. Colloidal Gold Immunoelectron Microscopy

After incubating for 6 h with medium, ETEC K88, or ETEC K88 plus *L. reuteri* LR1, as above, in the upper chamber of Transwell dishes, monolayers of IPEC-1 cells were fixed with 2.5% glutaraldehyde for 30 min and then dehydrated in a graded series of ethanol (30%, 50%, and 70%). The cells were transferred into epoxy resin Epon812 overnight and then heated for 72 h (each step of 35°C, 45°C, and 60°C for 24 h). Specimens were sectioned with a LKB-V ultramicrotome (LKB Bromma) and put on a nickel screen. The sections were treated with 0.5 mol/l NH_4_Cl for 15 min and then incubated in 3% hydrogen peroxide in the dark for 3 min. After blocking for 30 min using 5% BSA, the sections were incubated with a primary antibody (1 : 20 dilution) against zonula occluden-1 (ZO-1) or occludin (Cell Signaling Technology, Danvers, MA) overnight. The sections were then incubated with a colloidal gold-labeled secondary antibody (1 : 50 dilution) for 1 h. The sections were then stained with uranyl acetate and alkaline lead citrate for 15 min and visualized by transmission electron microscopy (Model H-7650, HITACHI, Japan).

### 2.5. Real-Time PCR

Total RNA was extracted using TRIpure reagent (Aidlab, Beijing, China). RNA quantity and quality were determined using a NanoDrop 1000 spectrophotometer (Thermo Fisher Scientific, Waltham, MA). The cDNA was generated using 1 *μ*g RNA with a PrimeScript RT reagent kit (Takara, Dalian, China). Real-time PCR was performed in triplicate using a CFX Connect detection system (Bio-Rad, Hercules, CA). The primers used for real-time PCR are listed in Table 1. Each reaction included 10 *μ*l iTaq Universal SYBR Green Supermix (Bio-Rad), 0.8 μl of each forward and reverse primers (10 μM), 2 μl 10-fold diluted cDNA, and 6.4 μL ddH_2_O. The thermocycler protocol consisted of 10 min at 95°C and 40 cycles of 30 s at 95°C, 30 s at 60°C, and 20 s at 72°C. GAPDH and *β*-actin were used as housekeeping genes. The relative transcript abundance was calculated using the 2^–ΔΔCt^ method, and the average of ΔCt (GAPDH) and ΔCt (*β*-actin) was used in this study. Treatment means were further normalized to the values obtained in the control treatment.

### 2.6. Western Blotting

Total protein was extracted from IPEC-1 cells using lysis buffer (KeyGEN, Nanjing, China) and clarified by centrifugation. Protein in supernatants was separated by 10% SDS-PAGE in a Bio-Rad system and transferred onto a polyvinylidene fluoride membrane (Bio-Rad). After blocking with 5% BSA for 30 min, the membranes were incubated overnight at 4°C with the appropriate primary antibodies for *β*-actin (Santa Cruz, CA), MLCK, phosphorylated MLCK, ZO-1, occludin, and F-actin (Cell Signaling Technology), followed by incubation with a horseradish peroxidase- (HRP-) conjugated secondary antibody for 1 h. Bands were detected using Clarity Western ECL Substrate (Bio-Rad). Band intensity was quantified using ImageJ software.

### 2.7. Statistical Analysis

Effects of treatment were assessed by one-way ANOVA using SPSS 16.0 software (SPSS Inc., Chicago, IL). Differences were considered to be significant at *P* < 0.05.

## 3. Results

### 3.1. *L. reuteri* LR1 Attenuated ETEC K88-Induced Damages in the Epithelial Barrier of IPEC-1 Cells

**Figure 1:**
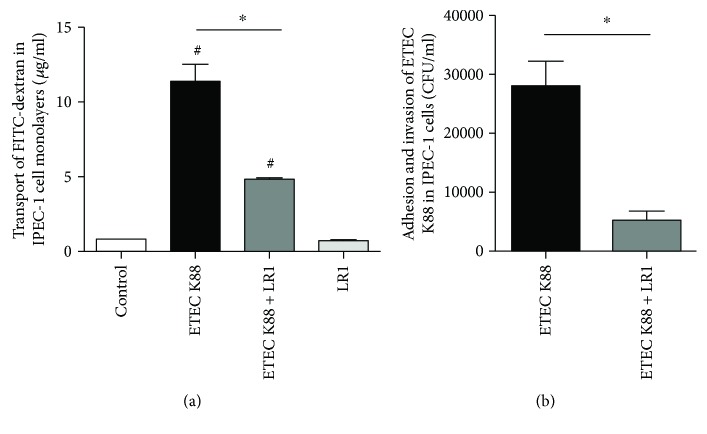
Permeability of IPEC-1 cell monolayers. (a) The transport of FITC-dextran across the IPEC-1 cell monolayers. (b) The adhesion and invasion of ETEC K88 in the IPEC-1 cell monolayers. All data are expressed as the mean ± SEM (*n* = 6) and representative of 3 independent experiments. Differences were determined by one-way ANOVA. ^#^*P* < 0.05 compared with control, ^∗^*P* < 0.05.

The permeability of IPEC-1 cell monolayers was determined using FITC-dextran. The transport of FITC-dextran in cells challenged with ETEC K88 was significantly increased when compared with the control (Figure 1(a)). Pretreatment with *L. reuteri* LR1 effectively decreased the transport of FITC-dextran in ETEC K88-infected IPEC-1 cell monolayers but had no effect when used alone. In addition, *L. reuteri* LR1 decreased the adhesion and invasion of ETEC K88 bacteria in the epithelial cells (Figure 1(b)). These results demonstrated that *L. reuteri* LR1 attenuated the ETEC K88-induced increase in the permeability of IPEC-1 cell monolayers.

### 3.2. *L. reuteri* LR1 Increased Gene Expression of TJ Proteins in ETEC K88-Infected IPEC-1 Cells

**Figure 2:**
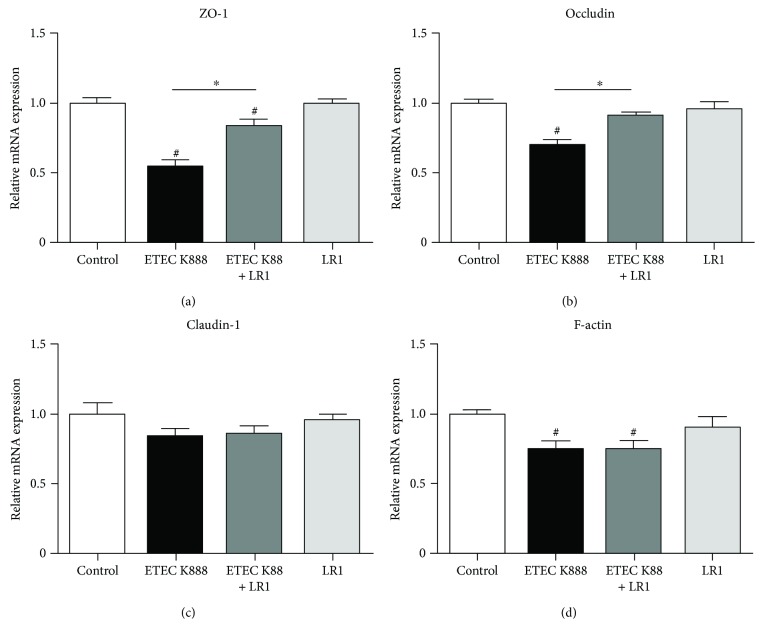
The mRNA levels of components of epithelial barrier in IPEC-1 cells. Relative transcript levels of ZO-1 (a), occludin (b), claudin*-1* (c), and F-actin (d) in IPEC-1 cells were determined by qPCR. GAPDH and *β*-actin were used as housekeeping genes, and the 2^–ΔΔCt^ method was used to determine the relative abundance. Data were further normalized to values measured in control treatments. All data are expressed as the mean ± SEM (*n* = 6) and representative of 3 independent experiments. Differences were determined by one-way ANOVA. ^#^*P* < 0.05 compared with control, ^∗^*P* < 0.05.

Challenge with ETEC K88 significantly decreased relative abundance of ZO-1 and occludin transcripts in IPEC-1 cells (Figures 2(a) and 2(b)). Pretreatment with *L. reuteri* LR1 again effectively offset this response to ETEC K88 resulting in much reduced decrease of ZO-1 and occludin expression. Neither ETEC K88 nor *L. reuteri* LR1 affected claudin transcripts in IPEC-1 cells (Figure 2(c)) while ETEC K88, but not *L. reuteri* LR1, decreased the expression of F-actin in IPEC-1 cells (Figure 2(d)).

**Figure 3:**
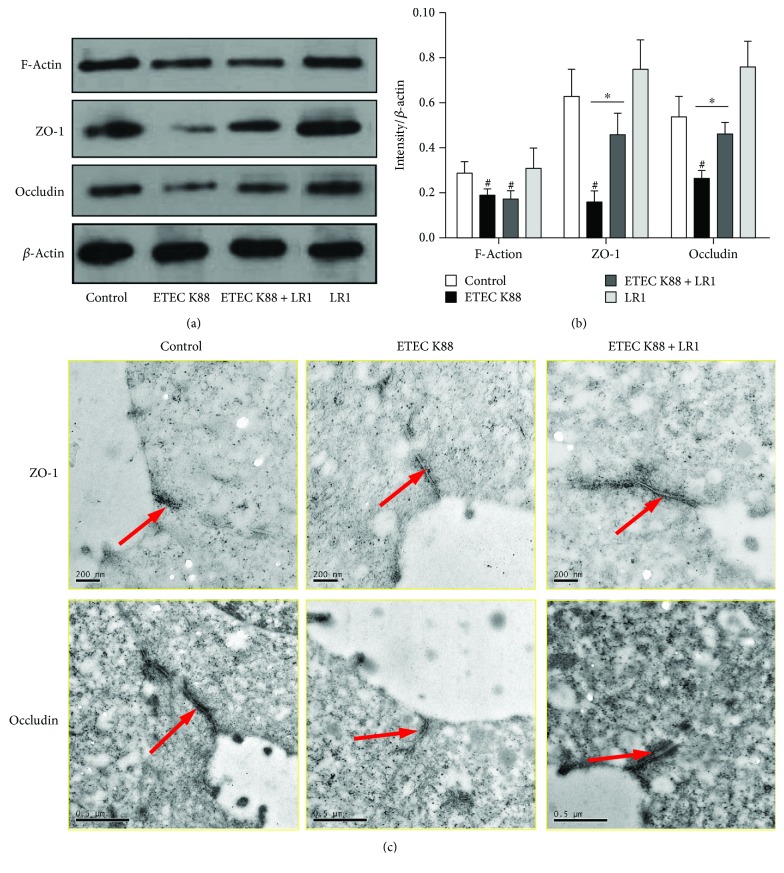
The contents of TJ proteins in IPEC-1 cells. (a) The contents of ZO-1, occludin, and F-actin in IPEC-1 cells were determined by Western blot analysis. (b) The relative intensity of target proteins to *β*-actin was calculated. All data are expressed as the mean ± SEM (*n* = 3) and representative of 3 independent experiments. Differences were determined by one-way ANOVA. ^#^*P* < 0.05 compared with control, ^∗^*P* < 0.05. (c) ZO-1 and occludin in IPEC-1 cells visualized by colloidal gold immunoelectron microscopy.

Western blotting was used to verify that the effects of *L. reuteri* LR1 during ETEC K88 challenge resulted in changes in protein content (Figure 3(a)). Consistent with the qPCR results, *L. reuteri* LR1 had no effect on the ETEC K88-induced decrease in F-actin protein, but it significantly increased ZO-1 and occludin protein content in ETEC K88-infected IPEC-1 cells (Figure 3(b)). These effects of *L. reuteri* LR1 on ZO-1 and occludin protein in ETEC-infected IPEC-1 cells were further confirmed by immunoultrastructural imaging (Figure 3(c)). Considered together, pretreatment with *L. reuteri* LR1 offset the gene expression and improved the status of the TJ proteins ZO-1 and occludin in IPEC-1 cells, otherwise challenged by ETEC K88.

### 3.3. LR1 Improved Expression of TJ Proteins in a MLCK-Dependent Manner

**Figure 4:**
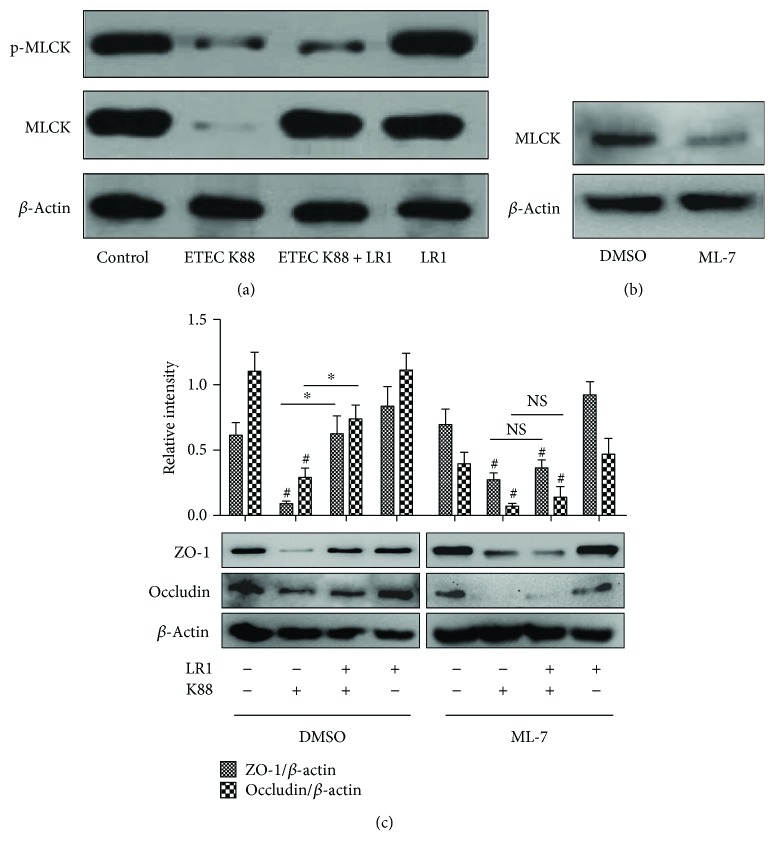
*L. reuteri* LR1 improved content of TJ proteins in a MLCK-dependent manner. (a) Content of MLCK and phosphorylated MLCK as influenced by ETEC K88 with and without *L. reuteri* LR1 in IPEC-1 cells, by Western blotting. (b) Inhibiting effect of the selective inhibitor ML-7 on the action of *L. reuteri* LR1 on MLCK in IPEC-1 cells. (c) Content of ZO-1 and occludin in IPEC-1 cells with ML-7 treatment. All data are expressed as the mean ± SEM (*n* = 6) and representative of 3 independent experiments. Differences were determined by one-way ANOVA. ^#^*P* < 0.05 compared with control, ^∗^*P* < 0.05.

The effects of *L. reuteri* LR1 in influencing the expression of the genes encoding TJ proteins and the proteins themselves were examined at the level of MLCK and phosphorylated MLCK in ETEC K88-infected IPEC-1 cells. Challenge with ETEC K88 decreased the cellular contents of MLCK and phosphorylated MLCK proteins (Figure 4(a)). Although *L. reuteri* LR1 treatment alone had no effect on phosphorylation of MLCK in ETEC K88-infected IPEC-1 cells, it diminished the extent of the decrease in MLCK protein caused by ETEC K88. The content of MLCK protein in IPEC-1 cells was suppressed by the selective inhibitor ML-7. It was then demonstrated that the increases in the content of ZO-1 and occludin caused by *L. reuteri* LR1 in ETEC K88-infected IPEC-1 cells were inhibited by ML-7. These data indicated that *L. reuteri* LR1 likely increased the expression of the TJ proteins via a MLCK-dependent pathway.

## 4. Discussion

Infection with the enterotoxigenic ETEC K88 usually impaired the integrity of the intestinal epithelial barrier [25, 26]. Although the regulatory mechanism of beneficial probiotics on the intestinal epithelial barrier is still unclear, it is established that some probiotics do improve barrier function, thereby maintaining intestinal homeostasis [27–30]. *L. reuteri* LR1, a new strain isolated from the feces of a healthy weaning piglet, exhibits both probiotic and functional properties in porcine intestinal epithelial cells [24]. It was therefore hypothesized that *L. reuteri* LR1 might protect against damage to epithelial barrier function in IPEC-1 cells, normally resulting from challenge with ETEC K88.

The present study clearly showed that *L. reuteri* LR1 substantially reduced the ETEC K88-induced increase in permeability of IPEC-1 cell monolayers and reduced the adhesion and/or colonization by the coliform. In addition, *L. reuteri* LR1 offset the extent of decreases in transcripts and proteins of TJ components ZO-1 and occludin provoked by ETEC K88 challenge in the IPEC-1 cells. Mechanistically, it was apparent that the protective effect of *L. reuteri* LR1 on ZO-1 and occludin was dependent upon the MLCK signaling pathway.

TJ proteins are the most important components of the intestinal epithelial barrier and play key roles in maintaining intestinal homeostasis by restricting the invasion of microbes and toxins [1, 22, 31]. The most important TJ proteins in the intestinal mucosa are ZO-1, occludin, and claudins [7], and the expression of all 3 in the intestine are decreased by infection with ETEC K88 [24, 30]. The present study showed that, for ZO-1 and occludin in IPEC-1 monolayers, this decrease from ETEC K88 infection was partially prevented by *L. reuteri* LR1. This finding is consistent with *L. reuteri* I5007 increasing expression of ZO-1 and occludin in similar intestinal porcine epithelial cells (IPEC-J2) [14]. Another probiotic, *L. plantarum*, ameliorated disruptions of ZO-1 and occludin in IPEC-J2 cells caused by ETEC K88 challenge [30]. Moreover, *L. acidophilus* and *Streptococcus thermophilus* attenuated decreases in ZO-1 expression in intestinal epithelial cells caused by enteroinvasive *E.coli* [32]. Similarly, *L. reuteri* I5007 increased claudin-1 expression in LPS-induced IPEC-J2 cells [14]. In contrast, in the present study, *L. reuteri* LR1 had no effect on claudin-1 expression in ETEC K88-infected IPEC-1 cells; perhaps the difference for claudin-1 might be due to a different strain of *L. reuteri* used. Nonetheless, the present study indicated that *L. reuteri* LR1 improved TJ protein expression in IPEC-1 monolayer infected by ETEC K88, consistent with *L. reuteri* LR1 improving integrity of the intestinal epithelial barrier.

The regulatory mechanisms of TJ proteins are complex. Myosin light-chain (MLC) phosphorylation can lead to increased intercellular permeability, and MLCK plays the key role in the regulation of MLC phosphorylation. A previous study showed that MLCK can inhibit apoptosis of intestinal epithelial cells [22, 33]. In the present study, ETEC K88 infection of IPEC-1 cells decreased protein levels of both MLCK and phosphorylated MLCK. Exposure to *L. reuteri* LR1 increased the levels of MLCK, but not those of phosphorylated MLCK, in ETEC-infected IPEC-1 cells. MLCK is known to regulate TJ barrier function [34]. The naphthalene sulfonamide ML-7 is a potent selective inhibitor of MLCK. A previous study in vascular endothelium showed that ML-7 could regulate TJ proteins ZO-1 and occludin expression through MLC phosphorylation and MLCK [35]. In the present study, ML-7 blocked the beneficial effect of *L. reuteri* LR1 on suppressed TJ proteins in IPEC-1 cells caused by ETEC K88, suggesting that *L. reuteri* LR1 likely acted through the MLCK signaling pathway.

Disruptions of TJ proteins are common in gut dysfunctions, such as postweaning diarrhea, reflux esophagitis, irritable bowel syndrome, and IBD [36]. For example, TJ proteins ZO-1 and occludin were decreased in irritable bowel syndrome [37, 38]. Recent studies have revealed that changes in TJ proteins could be potential biomarkers and targets for treatment, of these intestinal conditions [14, 19, 39]. Antibiotics, on the other hand, have little beneficial effect on disruptions of TJ proteins in intestinal diseases. Given the effectiveness of *L. reuteri* LR1 on TJ proteins demonstrated here, it might provide a safe and efficacious treatment strategy for many intestinal disorders caused by damage to the epithelial barrier.

In conclusion, *L. reuteri* LR1, in a MLCK-dependent manner, offset the decreased content of TJ proteins ZO-1 and occludin in IPEC-1 cells, otherwise caused by the damaging effect of ETEC K88 infection, suggesting that *L. reuteri* LR1 might offer promise for treating intestinal disorders with impaired function of the epithelial barrier.

## Figures and Tables

**Table 1:** Primers used for real-time PCR in this study.

Gene^1^	Primer sequence (5′-3′)	Accession number
ZO-1	Forward: AGCCCGAGGCGTGTTT	XM_013993251
	Reverse: GGTGGGAGGATGCTGTTG	
Occludin	Forward: GCACCCAGCAACGACAT	XM_005672525
	Reverse: CATAGACAGAATCCGAATCAC	
Claudin-1	Forward: GACTCCTTGCTGAATCTGA	NM_001244539
	Reverse: GCACCTCATCATCTTCCAT	
F-Actin	Forward: TCTGGAATGGTCGTTGGA	NM_001097454
	Reverse: CCTTGAATGTGGTGTCTGA	
*β*-Actin	Forward: CTGCGGCATCCACGAAACT	XM_003124280
	Reverse: AGGGCCGTGATCTCCTTCTG	
GAPDH	Forward: ACTCACTCTTCCACTTTTGATGCT	NM_001206359
	Reverse: TGTTGCTGTAGCCAAATTCA	

^1^zonula occluden-1

## Data Availability

The data used to support the findings of this study are available from the corresponding author upon request.
